# Pluripotent State Induction in Mouse Embryonic Fibroblast Using mRNAs of Reprogramming Factors

**DOI:** 10.3390/ijms151221840

**Published:** 2014-11-27

**Authors:** Ahmed Kamel El-Sayed, Zhentao Zhang, Lei Zhang, Zhiyong Liu, Louise C. Abbott, Yani Zhang, Bichun Li

**Affiliations:** 1 Provincial Key Laboratory of Molecular Design, College of Animal Science and Technology, Yangzhou University, Yangzhou 225009, China; E-Mails: ahmedkamelvet@gmail.com (A.K.E.-S.); yzuztzhang@gmail.com (Z.Z.); leizhang5807@gmail.com (L.Z.); liuyunfei198@gmail.com (Z.L.); 2 Department of Anatomy and Embryology, College of Veterinary Medicine, Suez Canal University, Ismailia 41522, Egypt; 3 Department of Veterinary Integrative Biosciences (VIBS), College of Veterinary Medicine and Biomedical Sciences, Texas A&M University, College Station, TX 77843-4458, USA; E-Mail: labbott@cvm.tamu.edu

**Keywords:** pluripotency, reprogramming, epigenetics, induced pluripotent stem cells, *in vitro* transcription

## Abstract

Reprogramming of somatic cells has great potential to provide therapeutic treatments for a number of diseases as well as provide insight into mechanisms underlying early embryonic development. Improvement of induced Pluripotent Stem Cells (iPSCs) generation through mRNA-based methods is currently an area of intense research. This approach provides a number of advantages over previously used methods such as DNA integration and insertional mutagenesis. Using transfection of specifically synthesized mRNAs of various pluripotency factors, we generated iPSCs from mouse embryonic fibroblast (MEF) cells. The genetic, epigenetic and functional properties of the iPSCs were evaluated at different times during the reprogramming process. We successfully introduced synthesized mRNAs, which localized correctly inside the cells and exhibited efficient and stable translation into proteins. Our work demonstrated a robust up-regulation and a gradual promoter de-methylation of the pluripotency markers, including non-transfected factors such as *Nanog*, *SSEA-1* (stage-specific embryonic antigen 1) and *Rex-1* (*ZFP-42*, zinc finger protein 42). Using embryonic stem cells (ESCs) conditions to culture the iPS cells resulted in formation of ES-like colonies after approximately 12 days with only five daily repeated transfections. The colonies were positive for alkaline phosphatase and pluripotency-specific markers associated with ESCs. This study revealed the ability of pluripotency induction and generation of mouse mRNA induced pluripotent stem cells (mRNA iPSCs) using transfection of specifically synthesized mRNAs of various pluripotency factors into mouse embryonic fibroblast (MEF) cells. These generated iPSCs exhibited molecular and functional properties similar to ESCs, which indicate that this method is an efficient and viable alternative to ESCs and can be used for further biological, developmental and therapeutic investigations.

## 1. Introduction

Great advances have been rapidly achieved in reprogramming of somatic cells to generate cells with greater pluripotency that aid in understanding the mechanisms of both differentiation and dedifferentiation. Numerous methods have been used for somatic cell reprogramming especially to the pluripotent state, which has been successfully achieved through transferring somatic cell nuclear material into oocytes (SCNT) [1,2,3] and through creation of cell hybrids by fusion of somatic cells with pluripotent cells [4,5,6]. In addition, reprogramming can be attained via exposing somatic cells directly to extracts of oocytes [7], embryonic germ cells [8], embryonic carcinoma cells or embryonic stem cells (ESCs) [9]. Although, there are significant technical and ethical challenges associated with the previously mentioned methods, it is clear that the cytoplasm of oocytes or pluripotent cells contain multiple factors responsible for reprogramming of somatic cells [1,10]. Recent stem cell genomic research generated induced pluripotent stem cells (iPSCs), suggesting that reprogramming of somatic cells can be achieved through ectopic expression of defined specific transcription factors (TFs) [11,12,13]. Utilization of iPSCs in science and medicine in place of ESCs eliminates the controversy of embryo utilization to derive stem cells, thereby overcoming the challenges of using non-ethical sources. iPSCs are produced by somatic cell reprogramming and are very similar to natural ESCs, showing the capacity to differentiate into numerous cell types and with the ability to self-renew. The possibility to derive iPSCs from a patient’s own cells also avoids the risk of immunologic rejection [13]. Moreover, it has possible broad application to solve problems in tissue engineering, regenerative medicine, cell replacement therapy and drug development. Since the initial generation of iPSCs from mouse embryonic fibroblast (MEF) cells by Takahashi and Yamanaka (2006) [11], there have been numerous refinements of this method as the potential therapeutic application of iPS cell lines generated by DNA-based approaches has been hampered by its modification of the host genome through the integration of DNA sequences that may cause mutations and/or activation of proto-oncogenes expression leading to malignancy and undesired results [13,14,15,16,17,18,19,20]. In spite of avoiding use of integrating viral vectors [11,21,22,23,24,25,26] and using the non-integrative DNA-based approaches including non-integrating viral vectors as adenovirus and sendai virus [27,28] or using virus-free approaches such as plasmids, minicircles and episomal vectors [14,15,29,30,31,32], the integration problem of DNA is difficult to be completely excluded. Therefore, discovery of more suitable ways for pluripotency induction without incurring genetic changes (*i.e.*, DNA-free methods) has become the focus of intense research efforts.

iPSCs have been derived through protein transduction of recombinant transcription factors [33,34] but the *in vivo* functional capacity of these bacteria-produced proteins may be compromised because essential modifications that only occur in mammalian cells may be lacking. In addition, post-translation modification of proteins may be a costly and low efficiency method. Also, over-expression and transfection of ESCs-associated microRNAs (miRNAs) were demonstrated to generate non-integrative human and mouse iPSCs [35,36,37] but a clear picture is needed of how miRNAs influence the pluripotent state of cells in order to render miRNA-based reprogramming an optimal and robust method. Recently, a safer and more efficient method for cellular reprogramming was performed through introduction of modified mRNA molecules encoding the reprogramming factors into somatic cells (mRNA-mediated gene delivery) and promoted highly efficient protein expression when used in hematopoietic progenitor cells, mesenchymal stromal cells, dendritic cell and lymphocytes [38,39,40]. Also, the transfected host cell undergoes a phenotypic conversion and steadily expresses the changed cell phenotype [41]. Using mRNA-mediated gene delivery, activated B cell and dendritic cells were able to express specific T lymphocyte responses when transfected with mRNAs of co-stimulatory molecules and viral antigens [42,43]. This method has been used to derive astrocytes from neurons; and fibroblasts as well as astrocytes that have been reprogrammed into cardiomyocytes [44]. This technique has been validated recently for human somatic cell reprogramming to generate iPSCs that showed successful activation of the pluripotency genes in the transfected somatic cells [45,46,47]. Warren, Mandal and their colleagues efficiently derived human iPSCs through long time exposure to a complex combination of modified RNA and immune suppressors [48,49]. In 2014, the derived human iPSCs using mRNAs under research-grade conditions were converted into a putative good manufacture practice (GMP) grade environment that represents a basis for the future use of human induced pluripotent stem cells (hiPSCs) in clinical trials [50].

The main goal of our research was to find suitable conditions for generation of mouse iPSCs by mRNA transfection of reprogramming factors into mouse somatic cells through minimum exposure time of transfection, to develop a model for further research, in spite of few reports concerning the utilization of mRNAs of reprogramming factors to induce pluripotency in murine species [51]. The generated iPSCs will be subjected to some analyses to determine whether their properties matched that of pluripotent embryonic stem cells. We also aimed to check the onset of marker genes activation through following up their expression levels and promoter methylation changes during reprogramming. This research will provide the basis for a better understanding of regulation of the reprogramming process and aid in discovering additional mechanisms of early embryonic developmental processes.

## 2. Results

### 2.1. Plasmid Construction and mRNA Synthesis

**Figure 1:**
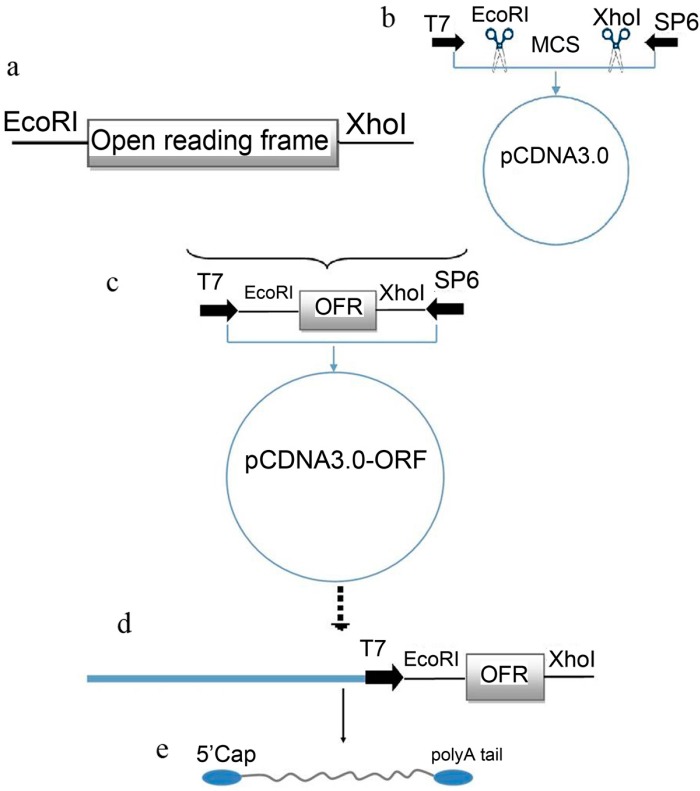
mRNA synthesis procedures. This diagram illustrates our protocol used to *in vitro* synthesize through cloning of the Open Reading Frame (ORF) of the interested genes (**a**); digestion the eukaryotic expression vector (pCDNA3) at the desired region (**b**); followed by the ligation of the produced products (**c**) in which the ORF was located downstream to the T7 promoter region. For mRNA synthesis, the newly constructed plasmid was linearized (**d**) through single cutting by *Xho*I. The linear plasmid was used for mRNA synthesis according to the manufacturer’s protocol (**e**).

**Figure 2:**
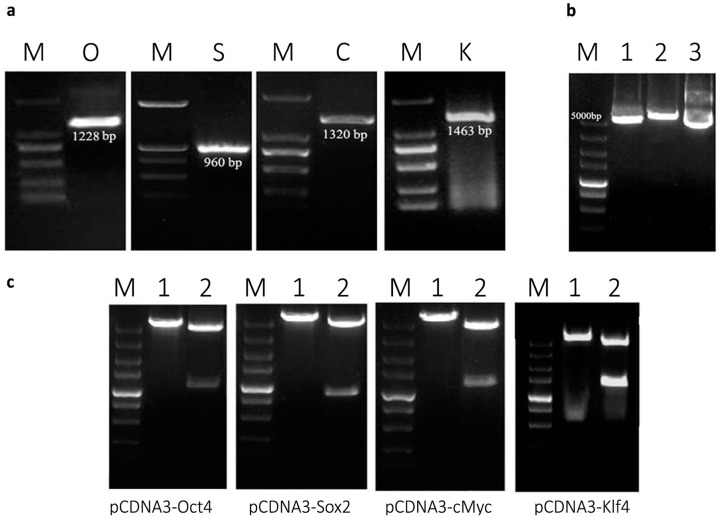
Plasmid construction and mRNA synthesis. (**a**) Successful amplification of the four factors from selected organs using specific primers for each. *Oct4* (O) and *Sox2* (S) were amplified from testes while *c-Myc* (C) was amplified from small intestine. *Klf4* (K) was correctly amplified from colon. The DNA marker (M) used was a 2000 bp marker; (**b**) The eukaryotic expression vector (pCDNA 3) was digested using the same enzymes used for cutting the genes. Lane (**1**) shows the result of double restriction of the plasmid using *Eco*RI and *Xho*I, while Lane (**2**) revealed the single cut using *Xho*I. Lane (**3**), shows the uncut circular plasmid. (M): DNA marker (5000 bp); (**c**) Confirmation of the successful cloning through digestion of the newly formed recombinant plasmids (pCDNA–*Oct4*, pCDNA–*Sox2*, pCDNA–*cMyc* and pCDNA–*Klf4*) using *Eco*RI and *Xho*I enzymes resulted in two bands that were similar to the expected size of each gene as showed in Lane (**2**), while Lane (**1**) shows the single cut of each recombinant plasmid (M): DNA marker (5000 bp).

The protocol of plasmid construction and mRNA synthesis was summarized as shown in Figure 1. The mammalian expression plasmid (pCDNA3) was used as a platform to prepare the mRNA of each transcription factor by *in vitro* transcription (IVT) using T7 RNA polymerase enzyme. Mouse organs expressing the four genes of interest (*Oct4*, *Sox2*, *c-Myc* and *Klf4*) were detected according to Mouse Genome Database (MGD) at the Mouse Genome Informatics website (MGI) (The Jackson Laboratory, Bar Harbor, Maine). Using RT-PCR, the four transcription factors were amplified from testis (*Oct4* and *Sox2*), small intestine (*c-Myc*), and colon (*Klf4*) using the previously mentioned protocol and primers (Figure 2a). The PCR products and the plasmid (pCDNA3) were purified, and then digested using restriction enzymes, *Eco*RI and *Xho*I (Figure 2b). The digested fragments and plasmid were purified, ligated to each other and then transformed into DH5α-*E.coli* competent bacterial cells. The positive clones for PCR were cultured for plasmid extraction. Extracted plasmids from positive colonies were single and double digested to demonstrate the correct expected fragment sizes of both pCDNA3 and the four genes (Figure 2c). For confirmation, the extracted recombinant plasmids were sent to Life Technologies Co., Ltd*.* (Shanghai, China) for sequencing which resulted in four specific fragments 1228, 960, 1320, and 1463 bp for *Oct4*, *Sox2*, *c-Myc* and *Klf4*, respectively. BLAST (Basic Local Alignment Search Tool) of the resulting sequences of the 4 products according to National Center for Biotechnology Information (NCBI, Bethesda, MD, USA) showed that each gene had a high percent of sequence homology to its corresponding reference sequence from mouse, as follows: 98%, 99%, 99% and 98% for *Oct4*, *Sox2*, *c-Myc* and *Klf4*, respectively.

To synthesize the mRNA of the inserted transcription factors, the designed recombinant plasmids were linearized using *Xho*I, then processed according to kit directions. After purification, the concentration and Optic Density (OD) values of each transcription factor mRNA were measured using a NanoDrop^®^ ND-1000 spectrophotometer (Thermo Scientific, Wilmington, DE, USA) as follows; 370.26, 434.97, 551.53, and 676.97 ng/µL for *Oct4*, *Sox2*, *c-Myc*, and *Klf4* respectively.

### 2.2. Optimization of Transfection Conditions

**Figure 3:**
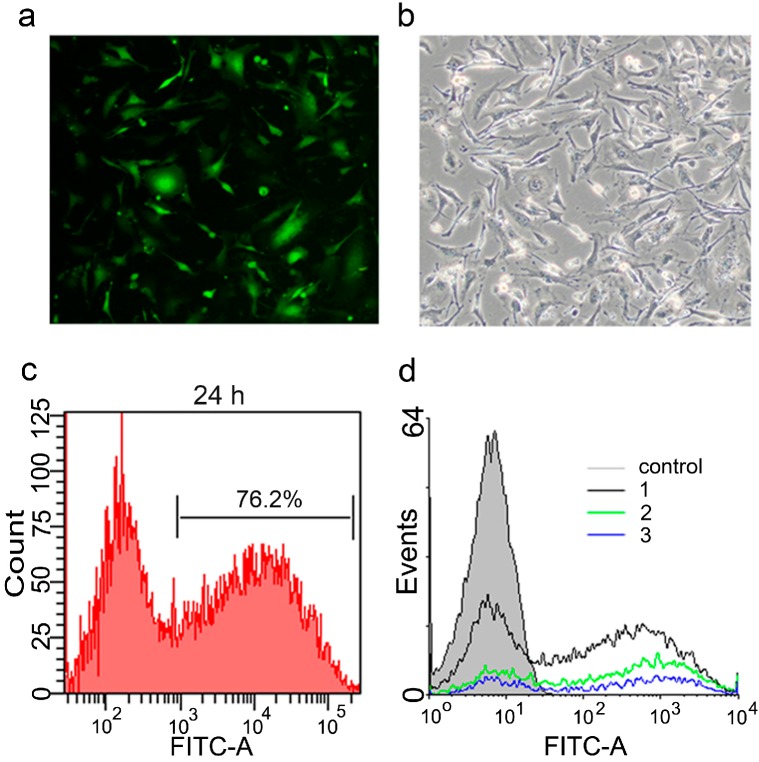
Transfection of mouse embryonic fibroblast (MEF) with green fluorescent protein (GFP) mRNA. Seeded cells in one well of a 24-well plate were transfected using 1 µg of mRNA of the green fluorescent protein according to the lipofection protocol (**a**,**b**); The microscopic fluorescence and bright field images (magnification 100×) of the cells 24 h after transfection, respectively; (**c**) Histogram for transfection efficiency analysis showed that more than 75% of the cells successfully expressed GFP after 24 h of lipofection; (**d**) Optimization of the transfected amount of mRNA revealed that 1 µg of mRNA (black line) achieved the highest expression value compared to 1.5 µg (green line) and 2 µg (blue line).

To optimize the transfection conditions, mRNA encoding green fluorescent protein (mGFP) was synthesized in the same manner mentioned above, and according to the lipofection protocol, it was transfected to MEF cells, which showed abundant expression after 24 h of transfection. More than 75% of the cells showed cytoplasmic localization of GFP (Figure 3). To determine the appropriate amount of mRNAs for transfection, the cells were transfected with different amounts of mRNA (1, 1.5 and 2 µg per each well), using 24-well culture plates. After 24 h, transfection efficiency was detected by Fluorescence Activated Cell Sorting (FACS), and we observed that the most suitable amount of mRNA was 1 µg per well as shown in Figure 3d.

**Figure 4:**
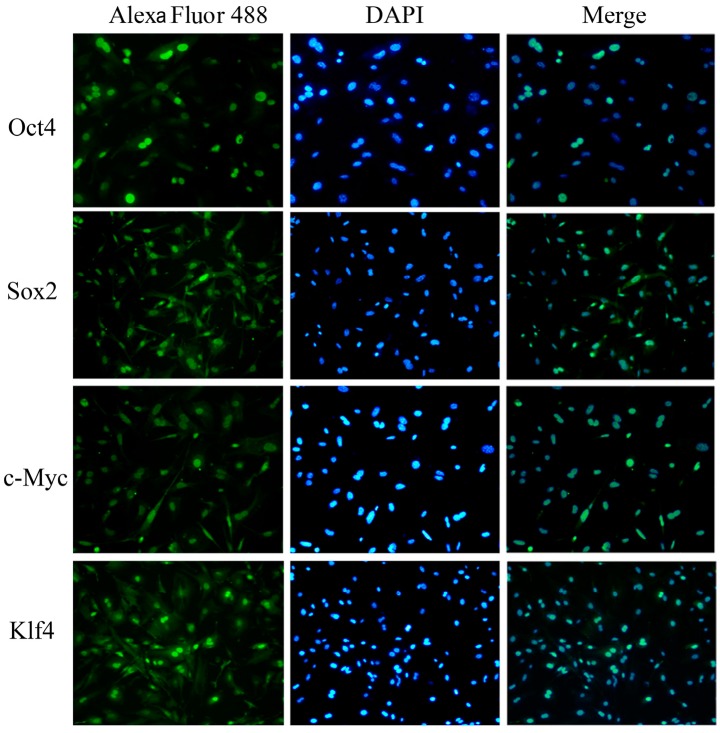
Transfection of MEF with mRNA of the four transcription factors. Cells were transfected with 1 µg mRNA (0.25 µg of each factor) and immunostained for the expressed proteins of the introduced factors after 24 h, showing nuclear localization of the 4 factors. Cellular nuclei were counter-stained by 4',6-diamidino-2-phenylindole (DAPI); (magnification 100×).

**Figure 5:**
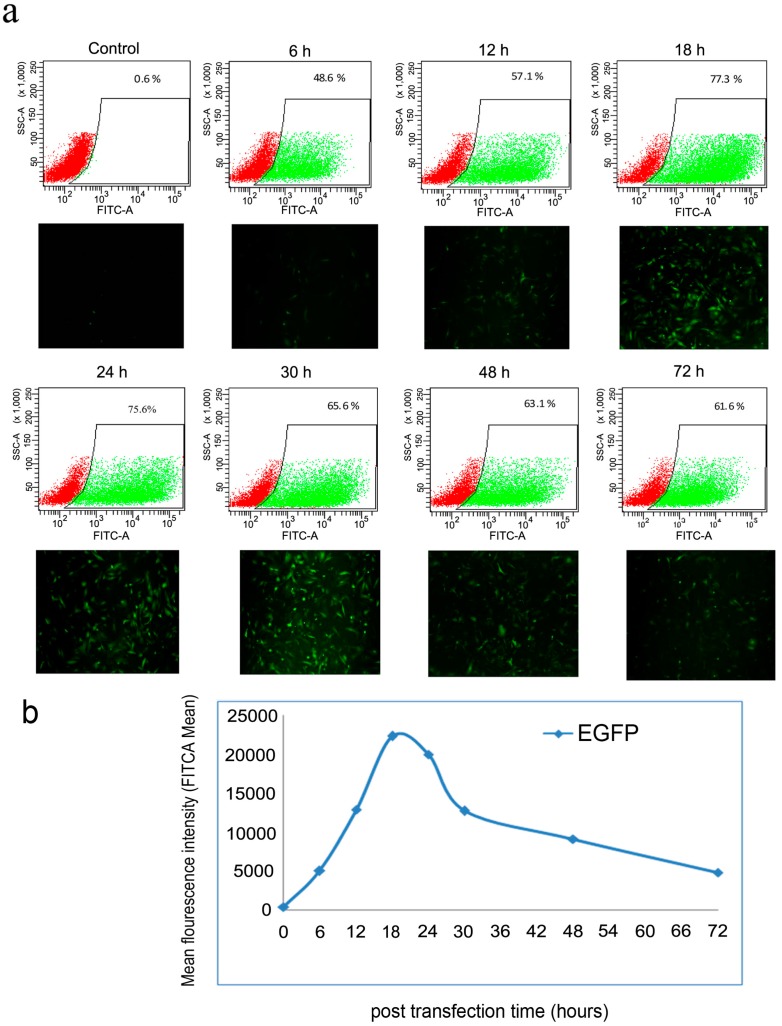
Kinetics and stability monitoring of the intracellular expressed proteins of the introduced factors. (**a**) Transfected cells with 1 µg of mRNA (GFP) were photographed under a fluorescence microscope (magnification 40×) and analyzed for fluorescence intensity at 6, 12, 18, 24, 48 and 72 h post transfection (green and red areas indicated the GFP- positive and GFP-negative cells, respectively); (**b**) Fluorescence Activated Cell Sorting (FACS) analysis revealed an increased intensity that reached the maximum expression at 18 h followed by decline until 72 h post transfection. Non-transfected MEF cells were used as a negative control.

Next, we transfected mRNAs of the reprogramming factors into cells using equal amounts of each factor; 0.25 µg of each mRNA were mixed together and transfected into the cells. One day later, the immunocytochemistry staining confirmed the correct, predominantly intra-nuclear localization of the reprogramming factors (Figure 4). To further characterize the reprogramming protocol, the kinetics and stability of the intracellular expressed protein after transfection was monitored by FACS using mRNA encoding the GFP. The results showed that protein expression could be detected after 6 h of transfection and reached maximum expression level at 18 h post transfection. Subsequently protein expression exhibited a rapid decline from 48 to 72 h, after which the lowest expression level persisted (Figure 5).

### 2.3. Generation of mRNA Induced Pluripotent Stem Cells (mRNA iPS)

**Figure 6:**
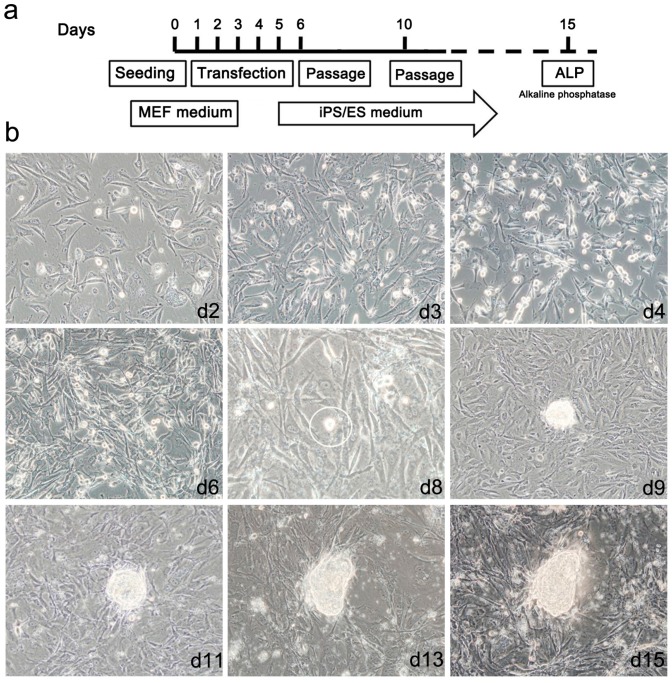
Generation of induced pluripotent stem cells. (**a**) Diagram illustrating the reprogramming protocol used in our experiment; (**b**) The lipofected cells showed changes in their morphology from the fibroblast appearance to round, like that of epithelial cells, which gradually increased in the first 7 days until the appearance of the first small colony-like structure at the 8th day of culture. The colonies subsequently increased in size to form embryonic stem (ES)-like colonies by the 15th day post transfection; (magnification 100×).

**Figure 7:**
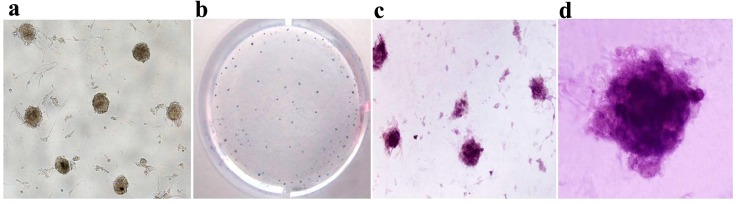

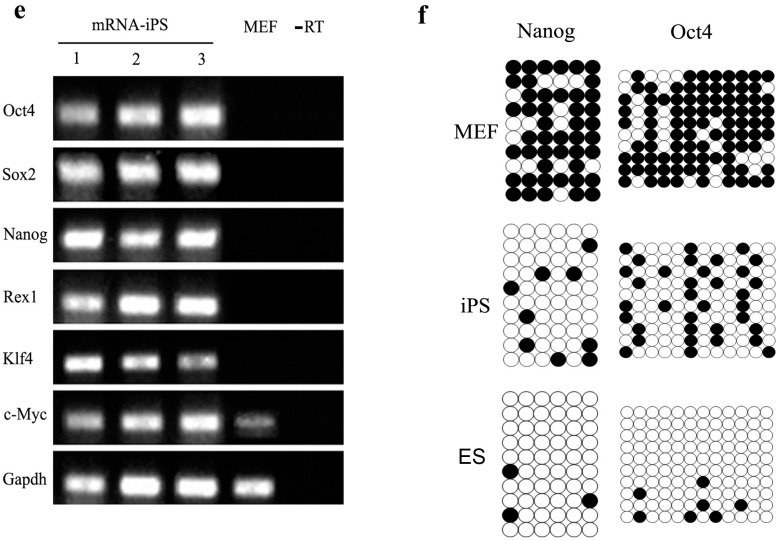
Characterization of the mRNA induced pluripotent stem cells (iPSCs). (**a**) The morphological characteristics of iPSCs with round ES-like colonies were distinguished by tightly defined borders and a high nuclear/cytoplasm ratio (magnification 40×); (**b**–**d**) showed positive alkaline phosphatase activity of the mRNA iPS either grossly (**b**, magnification 10×) or through microscopic observation (**c**, magnification 100×) and (**d**, magnification 200×); (**e**) Total RNA was isolated from three mRNA iPS clones and non-transfected MEF cells were used for RT-PCR to detect expression of pluripotency markers: *Oct4*, *Sox2*, *Nanog*, *Rex1*, *Klf4* and *cMyc*. Results showed that iPS colonies expressed all markers in contrast to MEF cells; (**f**) Detection of the methylation status of *Nanog* and *Oct4* promoters in both MEF cells, iPS and ESCs by bisulfite sequencing revealed a high percentage of de-methylation in both promoters compared to the parent MEF cells. Open circles indicate the un-methylated state and dark, filled circles indicate the methylated state.

According to the optimized transfection conditions, 1 × 10^5^ MEF cells were transfected with a mixture of 1 µg mRNA every 24 h according to the time schedule schematically illustrated in Figure 6a. Cells were subjected to 5 consecutive transfections followed by changing the culture conditions to that of ESCs. During transfection, we observed cellular morphological changes from the mesenchymal appearance of fibroblasts to compact, round epithelial cell morphology. These phenotypic changes increased until we observed small colony-like structures at day 8 of reprogramming. These colony-like structures increased in size and exhibited tightly defined borders and a high nuclear/cytoplasm ratio by day 15 after the first transfection (Figure 6b and Figure 7a). We obtained an average of 100 to 130 colonies per line of reprogramming with reprogramming efficiency about 0.1% to 0.13%.

### 2.4. Characterization and Identification of the Generated mRNA iPSCs

**Figure 8:**
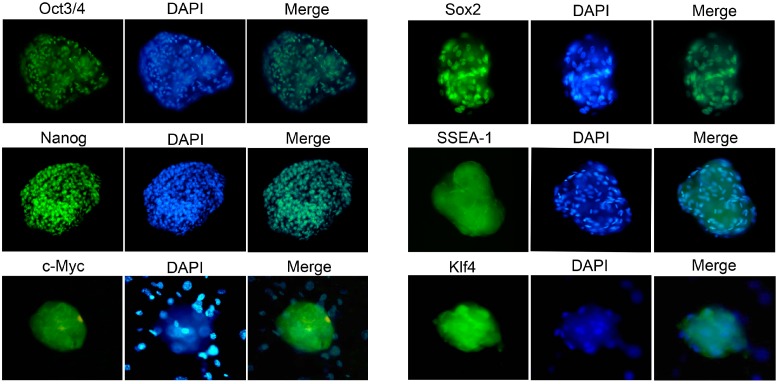
Immunofluorescence identification of the generated iPS. The newly derived mRNA iPS colonies were stained for the specialized markers of pluripotency: Oct4, Sox2, Nanog, SSEA-1, Klf4 and c-Myc. Nuclei were counter-stained with DAPI; (magnification 200×).

**Figure 9:**
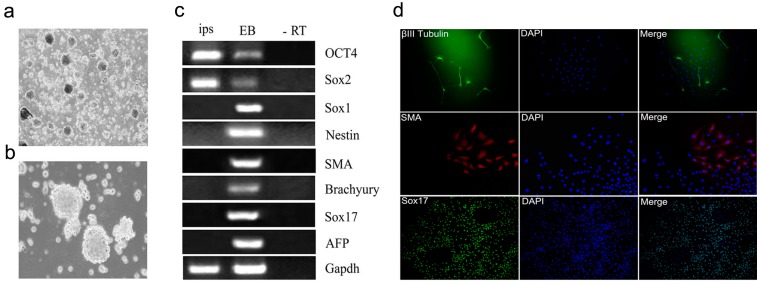

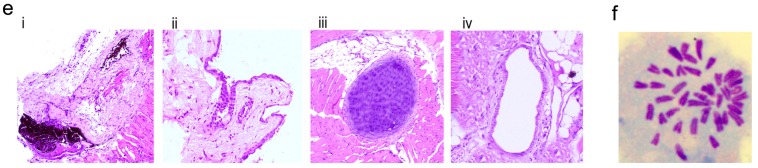
Pluripotency of the generated mRNA iPS from mouse MEF cells. (**a**,**b**) Assess pluripotency of the derived iPSCs; the cells were induced to form embryoid bodies (EB). The EB morphology in suspension culture (**a**, magnification 40×) (**b**, magnification 100×); (**c**) Comparative gene expression profiles of iPSCs and EB, using RT-PCR and showing that levels of pluripotent stem cell markers (Oct4, *Sox2*) decreased in EB while ectodermal markers (*Sox1*, *Nestin*), mesodermal markers (smooth muscle actin (*SMA*), *brachyury*) and endodermal markers (Sox17, AFP) were highly expressed in EB cells; (**d**) Immuno-staining confirming *in vitro* differentiation into three germ layers; βIII tubulin (ectodermal), SMA (mesodermal), and Sox17 (endodermal). Secondary antibodies were labeled with Alexa 488 (green, 40×) and Cy3 (red, 100×); (**e**) Hematoxylin and eosin staining of teratoma sections generated from mRNA iPSCs; (i) pigmented epithelium (ectoderm); (ii) epidermal tissue (ectoderm); (iii) cartilage like structure, fat and muscle tissues (mesoderm); (iv) epithelium (endoderm) 100×; (**f**) normal karyotyping and chromosomes numbers of mRNA iPSCs.

The generated mRNA induced pluripotent stem cells (iPSCs) colonies were subjected to a number of molecular and functional assays to assess pluripotency. The colonies were positive for alkaline phosphatase activity (ALP) (Figure 7b–d). The derived iPSCs colonies were stained for pluripotency proteins and were positive for Oct4, Sox2, Nanog, SSEA-1, Klf4, and c-MYC, while untreated cell populations were negative for these factors (Figure 8). To assure the pluripotency gene expression in the mRNA iPS cells, RT-PCR was conducted using fibroblast cells. The results demonstrated robust expression of pluripotency associated factors, *OCT4*, *Nanog*, *Rex-1*, *c-Myc*, and *Klf4*, compared to donor fibroblasts (Figure 7e). To confirm successful genomic reprogramming, the methylation patterns of mouse *Oct4* and *Nanog* gene promoter regions were analyzed in both the generated iPS clones and in the parental MEFs using the bisulfite conversion method mentioned above. Sequence analysis of the cloned promoter region revealed extensive de-methylation of the majority of analyzed Cytosine-phosphate-Guanine sites (CpGs) in iPSCs clones, while they remained methylated in MEF cells (Figure 7f). The developmental potential of the derived iPS was tested by determining its ability for *in vitro* differentiation. Embryoid bodies (EBs) were successfully formed and showed high expression of the specific markers for each of the three primary developmental germ layers using RT-PCR for detection of ectodermal markers (*Nestin* and *Sox1*), mesodermal markers (Smooth Muscle Aactin (*SMA*) and Brachyury) and endodermal markers (*Sox17* and Alpha feto protein (*AFP*)). Moreover, the results revealed lower expression of stem cell markers, including Oct4 and Sox2 (Figure 9a–c). The *in vitro* differentiation revealed also positive immune-staining for the specific markers of the three germ layers; βIII tubulin for ectoderm, smooth muscle actin (SMA) for mesoderm and Sox17 for endoderm (Figure 9d). To verify that the derived iPSCs had acquired pluripotency, they were subcutaneously injected into Severe Combined Immune deficient (SCID) mice and shown to form tumors after six weeks. Histological analysis revealed teratomas comprised of tissues of all three germ layers including cartilage, muscle, fat (mesoderm), pigmented epidermal tissue (ectoderm), and epithelium (endoderm) (Figure 9e). Karyotyping analysis of the iPS clones showed the normal karyotype and chromosome numbers of the murine species.

### 2.5. Genetic and Epigenetic Changes of the Reprogramming Factors and Pluripotency Markers during mRNA iPS Generation

**Figure 10:**
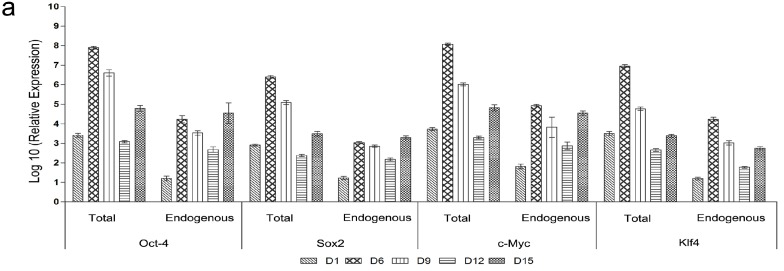

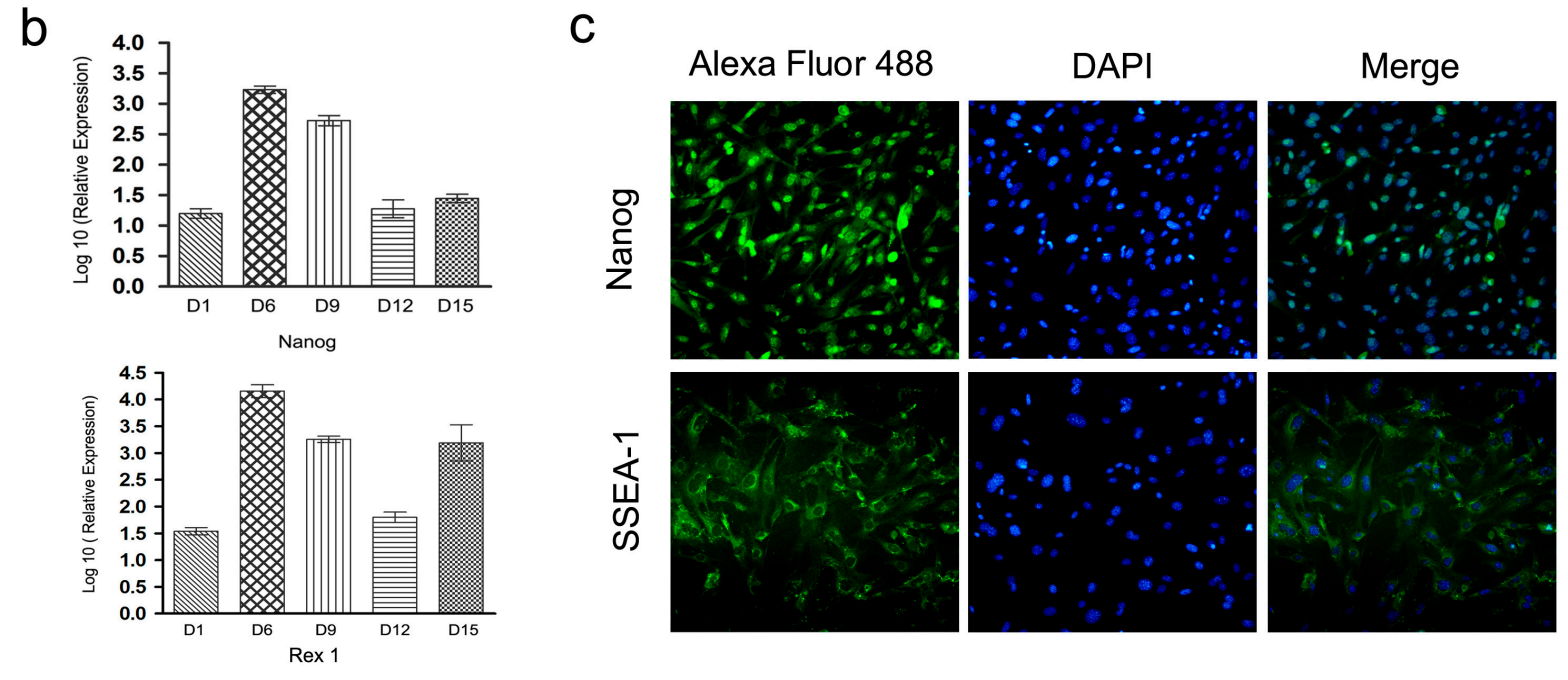
Changes in introduced factors and pluripotency markers during reprogramming. Quantification of pluripotency factors expression by qRT-PCR at different times (D1, D6, D9, D12 and D15) throughout the reprogramming timeline. (**a**) Total and endogenous expression levels of the transfected factors; *Oct4*, *Sox2*, *c-Myc* and *Klf4*; (**b**) the expression level of non-transfected factors (*Nanog* and *Rex-1*); (**c**) Immunostaining of non-transfected factors at day 6 confirmed the results of the qRT-PCR concerning *Nanog* and *SSEA-1*, as they showed nuclear localization of Nanog and surface expression of SSEA-1. Nuclei were counterstained with DAPI; (magnification 100×).

**Figure 11:**
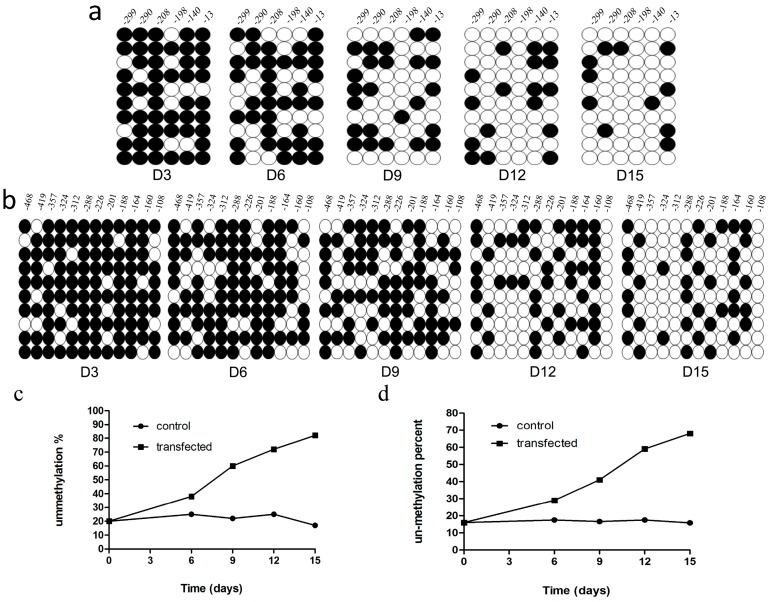
Epigenetic modification of the *Nanog* and *Oct4* promoters during the mRNA iPS generation. Methylation status of CpGs islands (columns) were analyzed from 10 randomly sequenced clones (rows) in the promoter region of *Nanog* (**a**) and *Oct4* (**b**) genes at different stages during reprogramming (D3, D6, D9, D12 and D15); Un-transfected MEF cells were used as control. Open circles indicate the un-methylated state and dark and filled circles indicate the methylated state; (**c**,**d**) The overall pattern and global analysis of the tested loci of *Nanog* and *Oct4* promoters, respectively.

The total and endogenous expression levels of the pluripotency genes including the reprogramming factors were quantified using the real time PCR at different times (D1, D6, D9, D12, and D15) during iPSCs generation. All transfected factors (*Oct-4*, *Sox2*, *c-Myc* and *Klf4*) were highly expressed after the first transfection while the non-transfected factors (*Nanog* and *Rex-1*) showed modestly increased expression. This period was followed by a robust increase in all factors’ quantities after the last transfection (D6). After that, expression levels of all factors decreased at D9 and D12 of reprogramming. At D15, pluripotency factor expression level increased (Figure 10a,b). To confirm the expression of non-transfected factors, cells were fixed after the last transfection and stained for Nanog and SSEA-1. Immunofluorescence analysis confirmed nuclear localization of Nanog and cell surface localization of SSEA-1 (Figure 10c). To detect the changes in the epigenetic status of the transfected cells, methylation patterns of the promoter regions were analyzed at different intervals of transfection (D1, D6, D9, D12 and D15). Gradual increases in de-methylation of *Oct4* and *Nanog* loci from D1 to D15 of reprogramming were observed. We observed that the percentage of non-methylation of the *Nanog* promoter changed from 20% at D0 to about 80% at D15 of the reprogramming time. The same changes were detected for the *Oct4* promoter, which revealed 70% un-methylation at D15 in contrast to the low percent (about 18%) prior to transfection (Figure 11).

## 3. Discussion

mRNA based gene delivery technology in cellular reprogramming provides new opportunities for biomedical research and clinical application. It can offer several advantages as a safe and highly efficient alternative to DNA-based and protein-mediated cellular reprogramming [52], and presents significant promise for use in clinical trials [53] as several studies have reported its use for immunotherapeutic purposes [54,55,56]. This technique completely eliminates genome manipulation and the DNA integration that may cause mutations and activation of proto-oncogenes expression, leading to possible malignancy and other undesired results. Several gene therapy applications prefer the use of mRNA instead of plasmid DNA and viral vectors in the face of its requirement for stable transgene expression [57]. mRNA reprogramming also surpasses obstacles in the protein delivery method including cost and time. mRNAs are directly translated into functional proteins in the cytoplasm with proper mammalian post-translational modifications; this results in greater functional efficiency when compared to the recombinant proteins produced in bacteria.

In our research using synthesis and preparation of mouse specific mRNA encoding reprogramming factors, we established efficient plasmids that could be used in a convenient manner for *in vitro* transcription and transfection. The amplified cDNAs of these factors were cloned in an expression vector (pCDNA3) downstream to the T7 promoter region. Our findings revealed sufficient establishment of the desired plasmids based on digestion results of the newly constructed vectors. The *in vitro* transcription reaction was incorporated with synthetic cap analog and also provided with poly (A) tailing reagents to promote efficient transfer of RNA in the cytoplasm in accordance to Yisraeli and Melton (1989) [58]. As it is known that exogenous single-stranded RNA (ssRNA) activates antiviral defenses in human cells [59,60,61,62,63], the immunogenic profile of synthetic RNA need to be reduced to allow repeated introduction of mRNAs in the target cells for a period of time long enough to induce reprogramming [48,64,65]. However, the probability of this immunogenic problem is not that pronounced in murine cells as mentioned by Tavernier (2012) [51] who showed successful onset of the reprogramming process in murine cells without any measure taken to suppress the innate immune response.

In this study, mRNAs of the transcription factors *Oct4*, *Sox2*, *Klf4* and *c-Myc* (OSKM) were successfully transfected into MEF cells with high expression level, in contrast to the low transfection efficiency and high cell mortality associated with use of plasmid DNA [45,66]. In addition, all factors were translated into their corresponding proteins that were properly localized in the nucleus of the transfected cells. This is the key exigency to perform its biological activity role in cellular reprogramming. Protein expression levels (Enhanced Green fluorescent Protein (EGFP) as indicator) were sustained for several days but then sharply decreased due to degradation of the transfected mRNA. Therefore, several repeated transfections of mRNA were required to preserve high levels of protein expression for prolonged time, which is necessary for cell reprogramming, in agreement with observations of Warren (2010) [48] and Jin (2011) [67]. To accomplish this, we used the cationic vehicle method for mRNA transfection as this facilitates its uptake from repeated transfections in concordance with the high reprogramming efficiency of Warren (2010) [48] and in comparison to the low efficiency resulting from electroporation [45,47]. Taking into account the cytotoxic effect of lipofectamine (LF), we minimized the exposure period to LF to as little as possible. With respect to pluripotency induction, our results demonstrated gradual cellular phenotypic changes similar to those observed by Chan (2009) [68] and Smith (2010) [69], which indicate the obvious roles of these transcription factors during stages of cell switching. We observed, during reprogramming, that the expression status of the embryonic stem cell genes passed through three stages. First, we observed potent up-regulation of the transferred factors (OSKM) during the transfection period presumably due to its proper expression and to stimulatory effects during activation of the endogenous factors. This was confirmed by up-regulation of the non-transfected pluripotency factors, Nanog, Rex1 and SSEA-1, which was consistent with the study of Yakubov (2010) [46] that reprogrammed the human fibroblasts. Loss of external stimuli due to stopping transfection resulted in a period of down-regulation for all these factors. Our findings in these two stages are similar to the findings of Plews and colleagues (2010) [45] who observed up-regulation of expression at day three and then decreased at day seven after cell electroporation. Also, Tavernier (2012) observed high expression levels of both transfected factors and pluripotency genes after only one transfection, which then significantly decreased in expression with increasing the time interval between consecutive transfections [51]. Our study revealed a third stage that represented a second up-regulation phase which suspected to be resulted from the activated endogenous genes of these factors at a time when most of the transferred factors were degraded. It was clear that our protocol has the ability to reactivate the inactive pluripotency genes, which was due to progressive de-methylation of promoter regions, resulting in turning on these factors. DNA methylation is considered to be a critical barrier to cellular reprogramming, so de-methylation at the promoter loci of pluripotency genes is a key epigenetic modification required for reprogramming, as mentioned by Simonsson (2004) [70]. This activation was accompanied by cell behavior alterations and produced small mouse ES-like colonies at D8, which was faster than obtained using DNA [11]. This may be attributed to the long time taken to transcribe the DNA in the nucleus, while mRNA was directly translated in cytoplasm.

The majority of the generated mRNA iPS colonies showed the same morphological characteristics of ESCs and expression of the typical pluripotency markers besides acquiring of characteristic pluripotency gene expression including Oct4, Sox2, Klf4, c-MYc, SSEA-1, Rex1 and Nanog; in addition to positive alkaline phosphatase (ALP) activity and its tri-lineage differentiation ability *in vitro* and teratoma formation *in vivo*. We also observed some extent of similarity to the methylation profile of the promoter regions of the key pluripotency genes. This acquisition of morphological and molecular properties of ESCs suggested that the synthesized mRNA-derived iPS clones were successfully reprogrammed.

The genomic integration approaches enable the stable expression of the factor genes in the host genome that explain their high iPSCs reprogramming efficiency. On the other hand, the previously applied non-integrating methods showed low reprogramming efficiency, ranging from 0.001% for the plasmids, minicircle DNAs, episomes and proteins delivery method to 0.1%–1% for the excisable vectors [71,72]. The mRNA-mediated gene delivery method achieved higher conversion efficiency in human [48], while in murine species, Tavernier and co-workers [51] succeeded only to activate the pluripotency genes in the transfected cells, without any detail about characterization of the colonies and efficiency of reprogramming. However, our study demonstrated the generation of iPSCs through only five consecutive transfections with moderate efficiency (about 0.1% to 0.13%) in comparison to the other non-integrating methods. We continue now to try multiple lines of reprogramming to achieve higher efficiency.

It was observed, in this study, that most of the transfected cells expressed pluripotency markers that were not part of the reprogramming cocktail (Nanog, SSEA1 and Rex1) at day 6, which confirmed the early stages of reprogramming into iPSCs [73]. Therefore, we will go on to study the effect of different culture conditions and small molecules on the maintenance of iPSCs to improve the final reprogramming efficiency and to further analyze molecular and functional properties of the generated mouse mRNA iPS.

## 4. Materials and Methods

### 4.1. RNA Extraction and cDNA Amplification

Total RNA was extracted using TRIZOL**^®^** reagent (15596-026, Invitrogen, Carlsbad, CA, USA) according to the manufacturer’s protocol. RNA quantification was performed using a NanoDrop 1000 Spectrophotometer (Thermo Scientific, Wilmington, DE, USA). 1 µg of isolated RNA was reverse transcribed to its corresponding cDNA using PrimeScript™ RT reagent Kit (Perfect Real Time, DRR037A, Takara, Dalian, China) according to the manufacturer’s protocol.

### 4.2. Plasmid Construction

The Open reading frames of Oct4, Sox2, Klf4 and c-Myc (OSKC) factors were amplified by PCR using PrimeSTAR^®^ Max DNA Polymerase (DR045A, Takara, Dalian, China) and primers sets (Table 1) in which the cutting sequences of *Eco*RI and *Xho*I enzymes were added to the 5' end of the forward and reverse primers respectively. The primers were synthesized by Life Technologies Co., Ltd. (Shanghai, China). The produced amplicons as well as the used expression vector (pCDNA3) were digested by *Eco*RI and *Xho*I then purified using an universal DNA purification kit (DP214, TIANGEN, Beijing, China) and finally ligated to each other using T4 DNA ligase enzyme (2011A, Takara, Dalian, China) according to the manufacturer’s protocol. The newly formed recombinant plasmids were cloned in DH5α-*E. coli* competent cells and re-extracted using TIAN prep Mini plasmid Kit (DP103, TIANGEN, Beijing, China).

**Table 1:** Primers used for amplification of the genes of interest. The restriction sites were underlined. *Eco*RI and *Xho*I restriction sites were used in forward and reverse primers, respectively. The underlined areas are the restriction sites of the used enzymes and the bolded are the protective nucleotides.

Gene	Accession No.	ORF Primers
*Oct4*	NM_013633	F: 5'**CG**GAATTC**CG**CCACCTTCCCCATGGCTGGACACC3' R: 5'**CC**CTCGAG**GG**TGATCAACAGCATCACTGAGCTTC3'
*Sox2*	NM_011443	F: 5'**CG**GAATTC**CG**ATGTATAACATGATGGAGACGGAGCT3' R: 5'**CC**CTCGAG**GG**TCACATGTGCGACAGGGGCAGT3'
*c-Myc*	NM_001177352	F: 5'**CG**GAATTC**CG**ATGCCCCTCAACGTGAACTTCACC3' R: 5'**CC**CTCGAG**GG**TTATGCACCAGAGTTTCGAAGC3'
*Klf4*	NM_010637	F: 5'**CG**GAATTC**CG**ATGAGGCAGCCACCTGGCGAGT3' R: 5'**CC**CTCGAG**GG**CTACGTGGGATTTAAAAGTGCCTC3'

### 4.3. In Vitro Transcription of mRNA

To synthesize the mRNA of the inserted transcription factors (TFs), the designed recombinant plasmids were linearized by *Xho*I, followed by *in vitro* RNA transcription (IVT) using the mMESSAGE mMACHINE T7 kit (AM1345, Ambion^®^, Grand Island, NY, USA) that allows 5' cap and poly (A) tail formation. The transcribed mRNA was then purified using MEGAclear™ Kit (AM1908, Ambion^®^, Grand Island, NY, USA) according to the protocol of the manufacturer. The mRNA concentration was measured using a NanoDrop 1000 spectrophotometer (Thermo Scientific, Wilmington, DE, USA).

### 4.4. Mouse Embryonic Fibroblast (MEF) Isolation

Procedures involving animals and their care conformed to the U.S. National Institute of Health guidelines (NIH Pub. No. 85-23, revised 1996) and all animal experiments were reviewed and approved by the Institutional Animal Care and Use Committee of School of animal Science and Technology, Yangzhou University and performed in accordance with the Regulations for the Administration of Affairs Concerning Experimental Animals (China, 1988) and the Standards for the administration of experimental practices (Jiangsu, China, 2008). Uterine horns from pregnant female mice (C57/BL) at 13 days post-coitum (d.p.c) were removed, washed with phosphate-buffered saline (PBS) and opened. Each embryo was separated from its placenta and surrounding membranes then the head and viscera were removed from the isolated embryos. The remaining parts were washed in PBS, minced using a pair of scissors until the pieces were able to be pipetted, then suspended in 0.25% Trypsin/ EDTA (Gibco^®^, Grand Island, NY, USA) solution (1–2 mL per embryo) and incubated at 37 °C for 15 min with gentle shaking. After trypsinization, an equal amount of MEF medium was added and pipetted up and down several times to dissociate the cells. The tissue/medium mixture was filtered to remove the remaining pieces of tissue, then the cells were collected by centrifugation (1000 rpm for 7 min) and resuspended in fresh medium. 10^6^ cells were cultured on 100 mm dishes at 37 °C with 5% CO_2_ (this is “passage No. 0”). We used MEFs within three passages to avoid replicative senescence.

### 4.5. Cell Culture

Mouse Embryonic fibroblasts (MEF) were cultured on gelatin coated dishes with MEF growth media containing high glucose Dulbecco’s Modified Eagle’s (DMEM) medium (CORNING, Corning, NY, USA) 10% FBS (Hyclone, South Logan, Utah, USA), 4 mM l-glutamine (25030-081, Gibco^®^, Grand Island, NY, USA) and 1:100 penicillin–streptomycin. After transfection, the transfected MEF cells and the generated iPSCs were maintained on a feeder layer of mitomycin-C inactivated MEF cells with mouse iPS/ES media containing DMEM (Gibco^®^, Grand Island, NY, USA), supplemented with 1000 U/mL Leukemia inhibitory factor (LIF, Sigma, St. Louis, MO, USA), 15% FBS (Hyclone, South Logan, UT, USA), 2 mM l-Glutamine (25030-08, Gibco^®^, Grand Island, NY, USA), 1 × 10^−4^ M non-essential amino acids (M7145, Sigma, St. Louis, MO, USA), 1 × 10^−4^ M 2-mercaptoethanol (MB0338, Bio Basic Inc., Amherst, NY, USA) and 1% penicillin and streptomycin.

### 4.6. Cell Transfection

Before transfection, the media was changed to prepared fresh media. RNA transfection was carried out through cationic lipid delivery vehicles using TransIT^®^–mRNA Transfection Kit (MIR2225, Mirus Bio, Madison, WI, USA). A total 1 µg of mRNA (0.25 µg of each transcription factor) was diluted in 100 µL of Opti-MEM followed by addition of 2 µL BOOST reagent and 2 µL TransIT^®^–mRNA. The complex was mixed gently, incubated at RT for (2–5 min), and then introduced to culture media. RNA transfection was performed in normal MEF media (DMEM + 10% FBS with antibiotic). The medium was changed 12 h after transfection to new media that did not contain transfection reagents.

### 4.7. Quantitative Real-Time PCR (qPCR)

The cDNA samples were analyzed by Real-Time PCR in a 7500 Real-Time PCR system (ABI, Carlsbad, CA, USA) using SYBR Premix Ex Taq™ (tli RNaseh Plus, RR420A, TAKRA, Dalian, China). Primer sequences are listed in Table 2. Relative quantification was calculated with 2^−ΔΔ*C*t^ and normalized to Gapdh. Data were presented as levels related to the expression level in MEF cells.

**Table 2:** Primers used for quantitative real time PCR (qPCR).

Gene		Forward Primer	Reverse Primer	References
*Oct4*	Total	5'CAGACCACCATCTGTCGCTTC3'	5'AGACTCCACCTCACACGGTTCTC3'	This study
	Endogenous	5'TCTTTCCACCAGGCCCCCGGCTC3'	5'TGCGGGCGGACATGGGGAGATCC3'	[11]
*Sox2*	Total	5'GGTTACCTCTTCCTCCCACTCCAG3'	5'TCACATGTGCGACAGGGGCAG3'	
	Endogenous	5'TAGAGCTAGACTCCGGGCGATGA3'	5'TTGCCTTAAACAAGACCACGAAA3'	
*c-Myc*	Total	5'CCTAGTGCTGCATGAGGAGACAC3'	5'TCCACAGACACCACATCAATTTCTT3'	This study
	Endogenous	5'TGACCTAACTCGAGGAGGAGCTGGAATC3'	5'AAGTTTGAGGCAGTTAAAATTATGGCTGAAGC3'	[11]
*Klf4*	Total	5'ACAGCCACCCACACTTGTGACTA3'	5'GGCGAATTTCCACCCACAG3'	This study
	Endogenous	5'GCGAACTCACACAGGCGAGAAACC3'	5'TCGCTTCCTCTTCCTCCGACACA3'	[11]
*Gapdh*	–	5'TGTGTCCGTCGTGGATCTGA3'	5'TTGCTGTTGAAGTCGCAGGAG3'	This study

### 4.8. Immunofluorescence

The cells were rinsed briefly with phosphate-buffered saline (PBS) and fixed for 20 min in 4% paraformaldehyde in 0.1 M phosphate buffer (pH 7.4) at room temperature. The cells were permeabilized for 10 min with 0.1% Triton X-100 in PBS, and blocked for 45–60 min with 4% bovine serum albumin in PBS at room temperature. Cells were incubated overnight at 4 °C with one of the following antibodies: anti-Oct4 (1:500; Abcam, Cambridge, MA, USA), anti-Sox2 (1:500; NB110-37235, Novus Biologicals, Littleton, CO, USA), anti-Nanog (1:500; Abcam, Cambridge, MA, USA), anti-c-Myc (1:250; bs-4963R, Bioss, Woburn, MA, USA), anti-Klf4 (1:250, bs-1064R, Bioss, Woburn, MA, USA). This was followed by incubation with the following secondary antibody: Alexa Fluor 488-labeled anti-rabbit IgG (1:500; Invitrogen, Carlsbad, CA, USA). Nuclei were counterstained using DAPI (1 mg/mL PBS; Invitrogen, Carlsbad, CA, USA).

### 4.9. Fluorescence Activated Cell Sorting (FACS)

To assess GFP positive cells, cultured cells washed twice with PBS after removing the culture media. Then the cells were detached with trypsin (0.05%, Gibco^®^, Grand Island, NY, USA) collected, centrifuged then re-suspended in PBS and were kept on ice until evaluation of GFP expression by a FACSAria Flowcytometer using FACSDiva software (Becton-Dickinson Immunocytometry Systems, BDIS, San Jose, CA, USA).

### 4.10. Alkaline Phosphatase Staining

Alkaline phosphatase (ALP) staining was performed using an AP staining kit (1101-050, SiDanSai, Beijing, China) according to the manufacturer’s protocol. Positive AP staining was recorded as blue to purple color.

### 4.11. Bisulfite Genomic Sequencing

Bisulfite treatment was performed using EZ DNA Methylation™ Kit (D5001, ZYMO RESEARCH CORP, Irvine, CA, USA) according to the manufacturer’s protocol. PCR primers are listed in Table 3. The amplified products were cloned into TOP10 (Vazyme Biotech, Nanjing, China). Ten clones were randomly selected, picked and sequenced with the M13 forward and M13 reverse primers for each gene (Invitrogen Co., Ltd., Shanghai, China).

**Table 3:** Primers used for DNA methylation (bisulfite sequencing).

Gene	Forward Primer	Reverse Primer
*Nanog*	5'GATTTTGTAGGTGGGATTAATTGTGAATTT3'	5'ACCAAAAAAACCCACACTCATATCAATATA3'
*Oct4*	F1: 5'GTTGTTTTGTTTTGGTTTTGGATAT3' F2: 5'ATGGGTTGAAATATTGGGTTTATTTA3'	5'CCACCCTCTAACCTTAACCTCTAAC3'

### 4.12. In Vitro Differentiation of mRNA iPSCs

Cells were chemically harvested by trypsinization and transferred to non-adherent bacteriological culture dishes in ES medium without Leukemia Inhibitory Factor (LIF) until formation of the aggregated cells of embryoid bodies was observed. Total RNA derived from plated embryoid bodies on day 6 was used for RT-PCR analysis for the three germ layer markers. The primers used for each germ layer are listed in Table 4. The cells were stained with anti-smooth muscle actin antibody (ab5694, Abcam, Cambridge, MA, USA), anti-Sox 17 antibody (cs-299, Santa Cruz, Dallas, TX, USA) and anti- βIII tubulin antibody (ab52901, Abcam, Cambridge, MA, USA). This was followed by incubation with the following secondary antibody: Alexa Fluor 488-labeled anti-rabbit IgG (A21206, Invitrogen, Carlsbad, CA, USA) or cy3^®^ anti-rabbit IgG (A10520, Invitrogen, Carlsbad, CA, USA). Nuclei were counterstained using DAPI (1 mg/mL PBS; Invitrogen, Carlsbad, CA, USA).

**Table 4:** Primers used for detection of the three germ layers in the formed Embryoid bodies.

Gene	Accession No.	Primers
*Sox1*	NM_009233	F: 5'GGATCTCTGGTCAAGTCGGAG3' R: 5'CTGGCGCTCGGCTCTCCAGAG3'
*Nestin*	NM_016701	F: 5' TCTGGAAGTCAACAGAGGTGG3' R: 5'ACGGAGTCTTGTTCACCTGC3'
*α-SMA*	NM_007392	F: 5'GAGAAGAGCTACGAACTGCCTGAC3' R: 5'CACATCTGCTGGAAGGTAGACAG3'
*Bra*	NM_009309	F: 5'GTTCCTGGTGCTGGCACCCTCTGC3' R: 5'CAGACCAGAGACTGGGATACTG3'
*Sox17*	NM_011441	F: 5'CACAGCAGAACCCAGATCTGCA3' R: 5'CATGTGCGGAGACATCAGCGGAG3'
*AFP*	NM_007423	F: 5'GTGAGCATTGCCTCCACGTGCTG3' R: 5'GTGACAGCCGCCAGCTGCTCCTC3'

### 4.13. Teratoma Formation and Histological Analysis

iPSCs were suspended at 1 × 10^7^ cells/mL of DMEM containing 10% FBS. 300 µL of the cell suspension were injected subcutaneously in SCID immune-deficient mice which were anesthetized with diethyl ether. Six weeks after injection, tumors were surgically dissected, fixed in 10% neutral formaldehyde, and embedded in paraffin. Sections were stained with hematoxylin and eosin.

### 4.14. Karyotyping Analysis

Karyotypes were determined following the Cold Spring Harbor Protocol [74] which is an adapted protocol from “Detection and Analysis of Mouse Genome Alterations and Specific Sequences,” Chapter 12, in Manipulating the Mouse Embryo, 3rd edition, Cold Spring Harbor Laboratory Press, Cold Spring Harbor, NY, USA.

## 5. Conclusions

In this study, mouse mRNAs of the key four reprogramming genes were successfully synthesized through their cloning in the eukaryotic expression vector pCDNA3.0 and successfully transferred to the somatic cells with efficient translation. Our work demonstrated a gradual de-methylation of the pluripotency markers promoters leading to their high expression during the reprogramming process. Five consecutive transfections resulted in pluripotency induction in the fully differentiated cells converting them into iPSCs with the same morphological, biological and functional properties of ESCs. The newly formed ES-like colonies were positive for alkaline phosphatase, expressed ES cell specific markers and showed an appropriate promoter methylation pattern typical of ESCs. Therefore, activation of mouse pluripotency genes and induced pluripotent stem cells generation can be achieved in a safe manner with better efficiency when using the mouse specific synthesized mRNAs for transfection.
